# Evaluation of potential miticide toxicity to *Varroa destructor* and honey bees*, Apis mellifera*, under laboratory conditions

**DOI:** 10.1038/s41598-020-78561-2

**Published:** 2020-12-09

**Authors:** Rassol Bahreini, Medhat Nasr, Cassandra Docherty, Olivia de Herdt, Samantha Muirhead, David Feindel

**Affiliations:** Plant and Bee Health Surveillance Section, Alberta Agriculture and Forestry, 17507 Fort Road NW, Edmonton, AB T5Y 6H3 Canada

**Keywords:** Biological techniques, Chemical biology

## Abstract

The honey bee, *Apis mellifera* L., is the world’s most important managed pollinator of agricultural crops, however, *Varroa* mite, *Varroa destructor* Anderson and Trueman, infestation has threatened honey bee survivorship. Low efficacy and development of *Varroa* mite resistance to currently used Varroacides has increased the demand for innovative, effective treatment tool options that exhibit high efficacy, while minimizing adverse effects on honey bee fitness. In this investigation, the toxicity of 16 active ingredients and 9 formulated products of registered miticides for use on crops from 12 chemical families were evaluated in comparison to amitraz on *Varroa* mites and honey bees using contact surface and topical exposures. It was found that fenpyroximate (93% mortality), spirotetramat (84% mortality) and spirodiclofen (70% mortality) had greater toxicity to *Varroa* mites, but high dose rates caused high bee mortality (> 60%). With this in mind, further research is needed to investigate other options to minimize the adverse effect of these compounds on bees. The results also found high toxicity of fenazaquin and etoxazole against *Varroa* mites causing 92% and 69% mortality, respectively; and were found to be safe on honey bees. Collectively, it is recommended that fenazaquin and etoxazole are candidates for a potential Varroacide and recommended for further testing against *Varroa* mites at the colony level.

## Introduction

In recent years, managed honey bees, *Apis mellifera* L., have had numerous health threats, which has resulted in losing a significantly higher annual loss of bee colonies averaging 30% or more when compared to long-term historical averages (15%)^1,2^. Although beekeepers are able to recoup their annual losses through colony management in the spring and summer, persistent high losses have made it more difficult and expensive to rebuild colony numbers. At the same time, high costs of buying bee packages or bees to replace dead outs, availability of healthy bees, increased cost of pest and disease management and unexpected business interruptions have created an unsustainable financial burden for beekeepers. In Canada^1^ and the USA^2^ bee colony losses were attributed to: bee parasitization by *Varroa* mites^3^, *Varroa destructor* Anderson and Trueman, *Nosema* spp.^4^, viruses^5^, poor nutrition^6^, and pesticides^7^. *Varroa* mites have been identified as the single most serious cause of colony loss and are considered the most prevalent honey bee pest in the world^8^. As an ectoparasite, *Varroa* mite threatens bee colony health by feeding on the fat bodies of immature and mature bees^9^. This weakens their immune system, uses the nutrient reserves required for overwintering in cold climates, and transmits viruses. The struggle to maintain the health of honey bees in the wake of *V. destructor* has resulted in the need to develop mite control options and to implement an integrated pest management approach that benefits beekeepers^10^. Tactics such as the use of genetically tolerant bees, cultural practices, and chemical control methods such as essential oils, organic acids, and synthetic miticides, are considered for mite management. Synthetic Varroacides, such as pyrethroids, organophosphate and formamidine, have been used to control *Varroa* mite populations globally, since the 1980’s. The long-term use of these products has led to the development of resistance in mite populations, the accumulation of residues in bee products, and an increased risk to human health^11–13^. Currently, populations of *Varroa* mites globally have developed resistance to the pyrethroid, Apistan (824 mg of tau-fluvalinate/strip, Vita Europe), and to the organophosphate, Checkmite (1300 mg coumaphos/strip, Bayer)^1^. Additionally, resistance is emerging to the only remaining viable formamidine miticide, Apivar (500 mg of amitraz/strip, Veto Pharma)^12^. This new reality highlights the need for alternative synthetic products with different modes of action, which would support an integrated approach to the management of mites and maintenance of healthy bees. In an attempt to manage resistance to synthetic miticides, organic acids such as formic acid, oxalic acid and essential oil-based miticides have been used in rotation; however, mite control has been variable. The reluctance of beekeepers to use organic acid and essential oil-based miticides has led to an increased reliance on synthetic miticides. Therefore, it is imperative to find new active ingredients (AIs) with modes of action different from currently available miticides that will selectively kill *Varroa* mites without harming honey bees.

Pesticide resistance testing from other insect species can be adapted to test novel chemicals on *Varroa* mites and honey bees. Plapp et al*.*^14^ initially developed a glass vial method to evaluate the presence of pesticide resistance in the tobacco budworm moth, *Heliothis virescens* (F.) in cotton. In this technique, synthetic miticides were dissolved in a solvent and applied to cover the inner surface of 20 mL borosilicate glass scintillation vials. This method was also used for determining the resistance or toxicity of chemicals against red-legged earth mites^15^, *Halotydeus destructor* (Tucker), two spotted spider mites^16^, *Tetranychus urticae* Koch, silverleaf whiteflies^17^, *Bemisia argentifolii* Bellows and Perring, plant bugs^18^, Thrips^19^*,* as well *Varroa* mites^20–22^. In this study, a modified glass vial technique was adopted form miticide screening^22^ and *Varroa* resistance^23^ studies. Determining the median lethal concentration (LC_50_), dose (LD_50_) and mortality rates of *Varroa* mites and honey bees to candidate compounds using bioassay techniques are standard approaches for evaluating the efficacy and toxicity of miticides on animals. These methods are also used to monitor the degree of resistance in *Varroa* mite populations. Vandervalk^22^ found high toxicity when exposing *Varroa* mites to acequinocyl followed by clofentezine, spiromesifen and bifenazate using the glass vial technique. Ali et al*.*^24^ reported a high mite mortality for tebufenpyrad, however, this product exhibited a high bee mortality. Fenpyroximate caused significantly higher mortality in queens (at 9.7 µg/queen), compared to worker bees^25^, but according Leite et al*.*^26^, fenpyroximate at 1000 mgL^−1^ was safe for bees. Recently, based on an RNAi method, Ziegelman et al*.*^27^ found that lithium chloride used to precipitate RNA was effective at controlling *Varroa* mites under laboratory conditions. The study demonstrated that 10.6 µg of lithium chloride fed to bees killed 100% of mites during a 48 h observation. Maggi et al*.*^28^ reported the LC_50_ for susceptible *Varroa* mite population in Argentina (0.1 μg/dish). In Canada, amitraz is one of the most commonly used in-hive miticides, however, the LC_50_ baseline levels for amitraz and other Varroacides, for susceptible and resistant *Varroa* mite populations, have not been determined.

The objective of this investigation was to develop laboratory bioassay techniques for the screening of potential miticides against *V. destructor*, and to determine the toxicity of 16 miticidal AIs and 9 commercially available formulated miticidal products (FPs) from 12 different chemical families in comparison to amitraz to *V. destructor* and *A. mellifera* (Supplementary Table S1).

## Results

### Solvent, reference and death controls

Acetone, acetonitrile and water were chosen as solvents in the pre-test for making dilutions, and tested as solvent controls for AIs and FPs, respectively. Acetonitrile was used as a specific solvent for clofentezine; however, other AIs (n = 16) were dissolved in acetone to make a stock solution. All FPs (n = 10) were diluted in water. The solvents did not have a significant influence on mite or bee mortality, with the exception of acetonitrile which resulted in a higher mite and bee mortality (5–35%) compared to other solvents (0–11%) (Supplementary Table S2). Amitraz and Mitaban were used as reference control for AIs and FPs, respectively. Amitraz killed ≥ 90% of *Varroa* mites when the mites were exposed to a dilution of ≥ 10 mgL^−1^ in surface contact (glass vial) (F = 40.67; df = 6, 21; p < 0.0001); or ≥ 68% in dilution ≥ 0.000117 µg/mite in topical (micro-applicator) (F = 756.89; df = 6, 21; p < 0.0001) methods. Mitaban, however, achieved > 32% mite mortality at ≥ 1000 mgL^−1^, significantly higher than other FPs and the control (F = 8.61; df = 5, 18; p = 0.0003). Amitraz was safe on newly-emerged worker bees when treated with ≤ 100 mgL^−1^ by surface contact (Mason jar) (F = 18.91; df = 6, 21; p < 0.0001), or ≤ 0.78 µg/bee by topical applications (F = 43.96; df = 6, 28; p < 0.0001). In the surface contact method, Mitaban in dilution ≤ 0.1 mgL^−1^ killed ≥ 76% bees (F = 86.95; df = 6, 27; p < 0.0001), compared to ≤ 3% mortality at ≤ 0.78 µg/bee in topical applications (F = 2.31; df = 6, 27; p = 0.0629) (Supplementary Tables S3, S4). In the investigation, dimethoate (0.000033 mgL^−1^) and amitraz (100,000 mgL^−1^) were used as death controls to verify the sensitivity of *A. mellifera* and *V. destructor* to selected compounds, respectively. Both treatments resulted in 100% mortality of bees and mites. Thus, mortality variables of dimethoate and amitraz dilution of 100,000 mgL^−1^ were removed from the statistical analyses.

### Toxicity assay on *Varroa* mites

#### AIs surface contact toxicity assay

**Figure 1:**
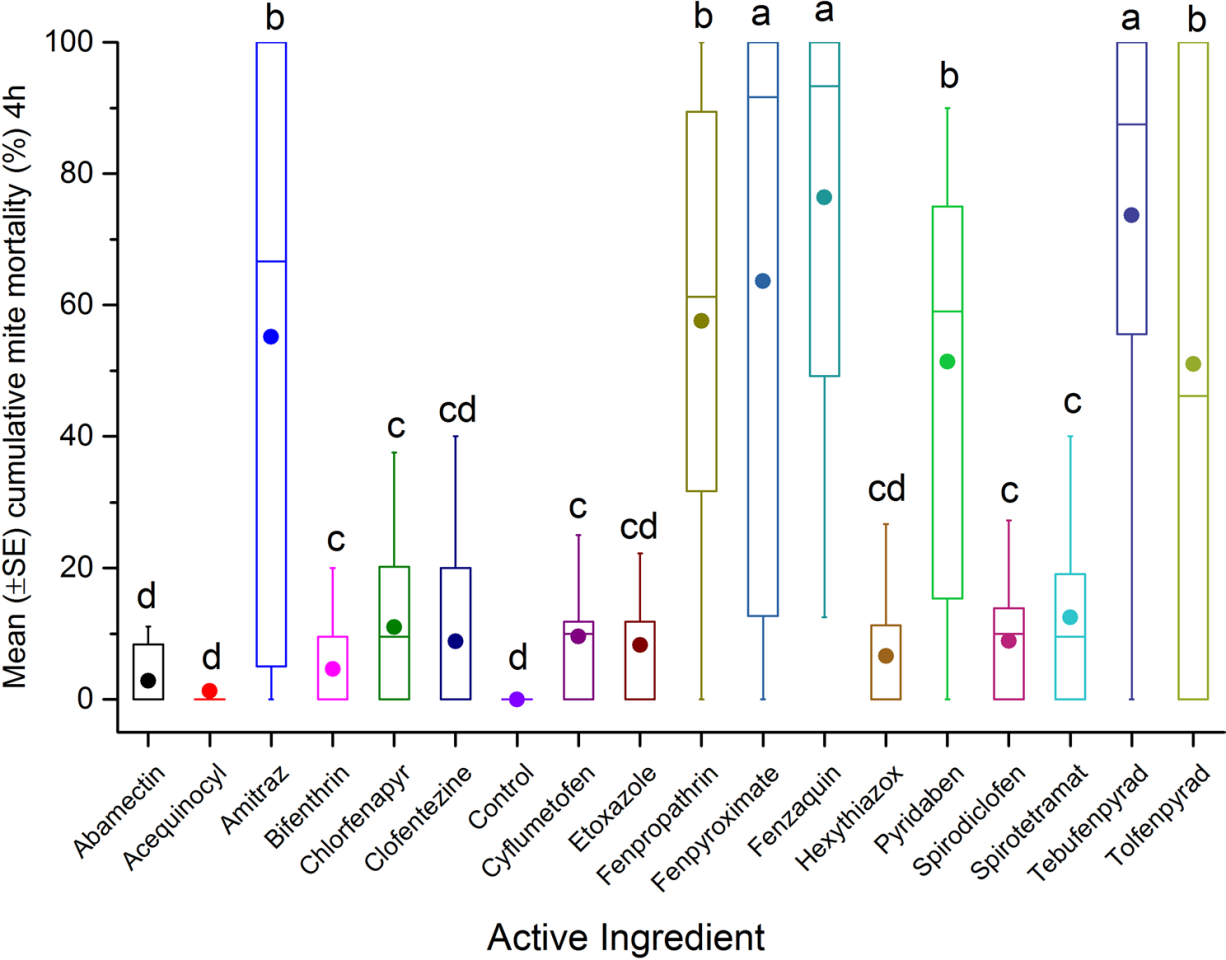
Mean (± SE) cumulative mite mortality (%) during 4 h exposure to different dilutions (0.1 mgL^−1^, 1 mgL^−1^, 10 mgL^−1^, 100 mgL^−1^, 1,000 mgL^−1^, or 10,000 mgL^−1^) of tested AIs in acute surface contact toxicity assay using 20 mL borosilicate glass scintillation vials. A group of mites was treated with different dilutions of amitraz as reference control, or left untreated (control) as negative control. The boxplots present the standard error (length of box), mean (solid circle), median (horizontal line), 5th and 95th percentiles (lower and upper vertical lines). Each box indicates average mortality for replications of each AI (n = 24) or control (n = 4). Means with the same letter among treatments are not significantly different (*p* > 0.05).

**Figure 2:**
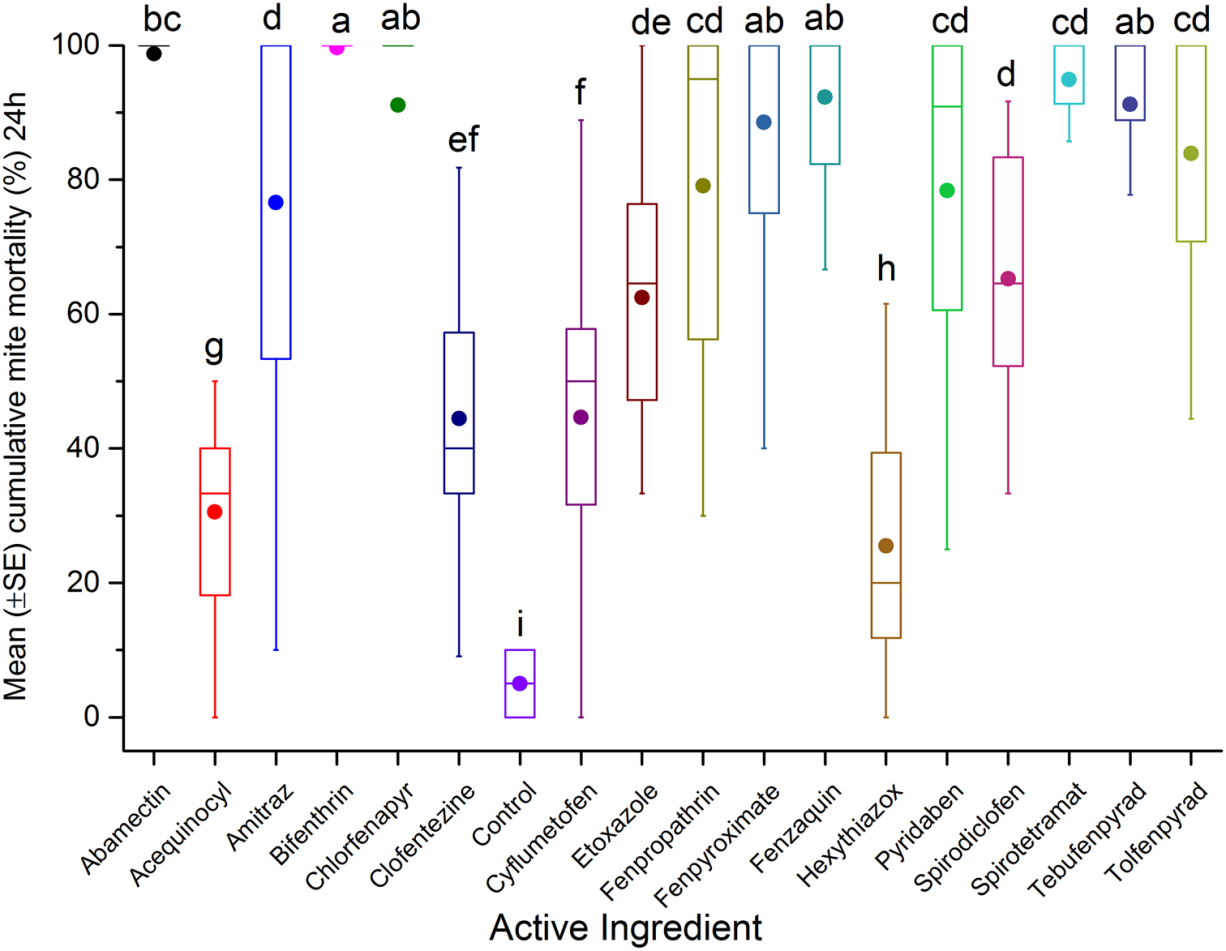
Mean (± SE) cumulative mite mortality (%) during 24 h exposure to different dilutions (0.1 mgL^−1^, 1 mgL^−1^, 10 mgL^−1^, 100 mgL^−1^, 1000 mgL^−1^, or 10,000 mgL^−1^) of tested AIs in acute surface contact toxicity assay using 20 mL borosilicate glass scintillation vials. A group of mites was treated with different dilutions of amitraz as reference control, or left untreated (control) as negative control. The boxplots present the standard error (length of box), mean (solid circle), median (horizontal line), 5th and 95th percentiles (lower and upper vertical lines). Each box indicates average mortality for replications of each AI (n = 24) or control (n = 4). Means with the same letter among treatments are not significantly different (*p* > 0.05).

AIs (n = 17) were tested to determine the surface contact toxicity on *Varroa* mites by coating the AIs on the inner surface of glass vials. Initial results show high variability in toxicity after 4 h exposure (F = 25.92; df = 17, 393; p < 0.0001) (Fig. 1). The highest cumulative mortalities were recorded when mites were exposed to fenazaquin (76.4 ± 5.2%), tebufenpyrad (73.7 ± 5.3%) and fenpyroximate (63.6 ± 5.2%) compared to the reference control, amitraz (55.1 ± 5.2%). At 4 h exposure, cumulative mite mortality for fenpropathrin (57.6 ± 5.2%), pyridaben (51.4 ± 5.2%) and tolfenpyrad (51 ± 5.2%) treatment were not significantly different from amitraz. However, after 24 h post exposure, cumulative mite mortality was greatest for bifenthrin (94.3 ± 3.5%), fenpyroximate (93.2 ± 2.5%), fenazaquin (92.3 ± 4.3%), tebufenpyrad (91.2 ± 4.5%), chlorfenapyr (88.9 ± 3.1%) and abamectin (88.9 ± 3.1%). All AIs listed above had significantly higher cumulative mite mortality, compared to amitraz (76.6 ± 4.3%) and others AIs (F = 31.49; df = 17, 388; p < 0.0001) (Fig. 2). Results also showed a relatively low cumulative mite mortality for clofentezine after 4 h (8.8 ± 5.2%) exposure or after 24 h (61.5 ± 3.1%) post-treatment in comparison to amitraz. In this case, mite mortality was likely attributed to the solvent acetonitrile (Supplementary Table S2).

#### FPs surface contact toxicity assay

**Figure 3:**
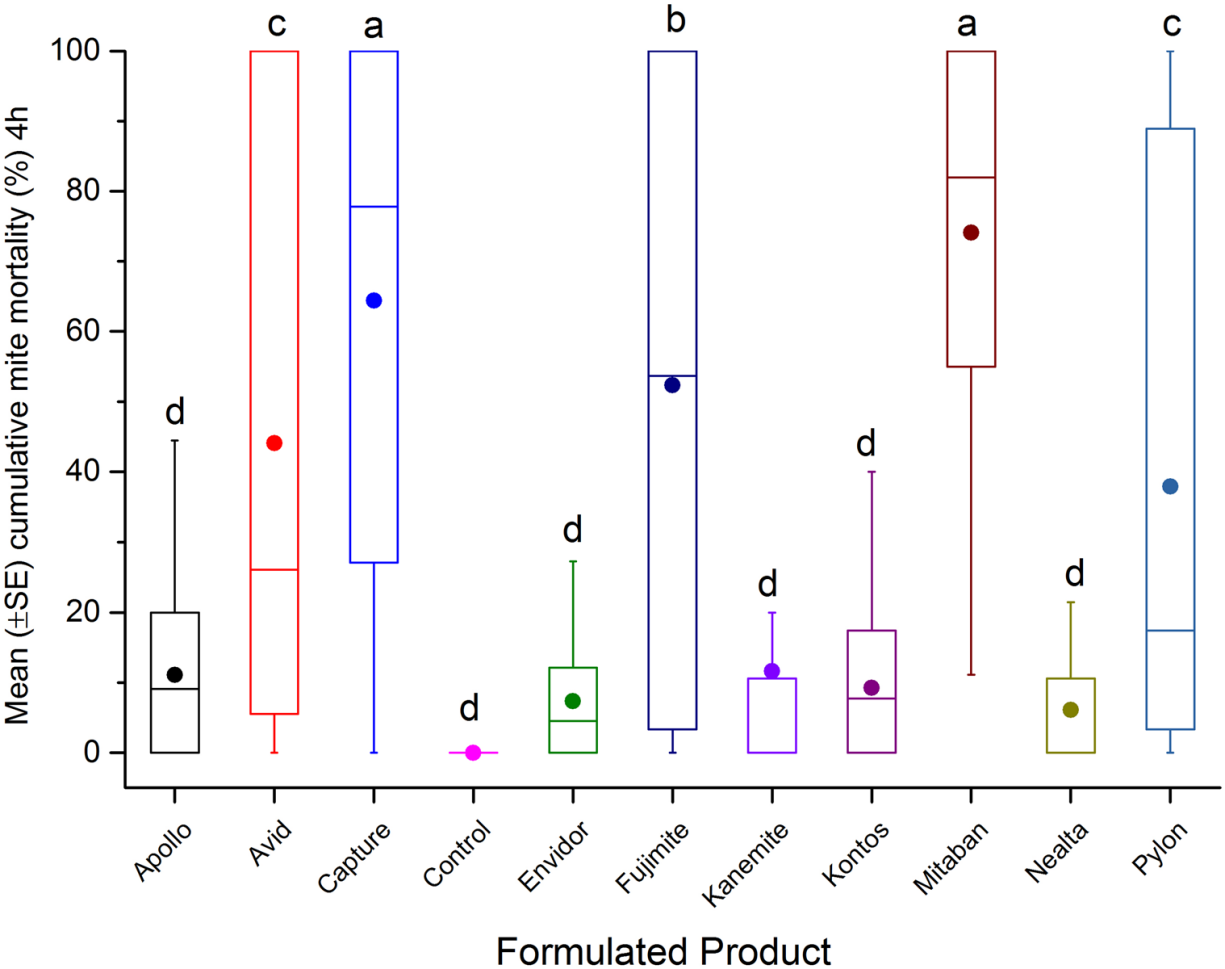
Mean (± SE) cumulative mite mortality (%) during 4 h exposure to different dilutions (0.1 mgL^−1^, 1 mgL^−1^, 10 mgL^−1^, 100 mgL^−1^, 1000 mgL^−1^, or 10,000 mgL^−1^) of tested FPs in acute surface contact toxicity assay using 20 mL borosilicate glass scintillation vials. A group of mites was treated with different dilutions of Mitaban as reference control, or left untreated (control) as negative control. The boxplots present the standard error (length of box), mean (solid circle), median (horizontal line), 5th and 95th percentiles (lower and upper vertical lines). Each box indicates average mortality for replications of each FP (n = 24) or control (n = 4). Means with the same letter among treatments are not significantly different (*p* > 0.05).

**Figure 4:**
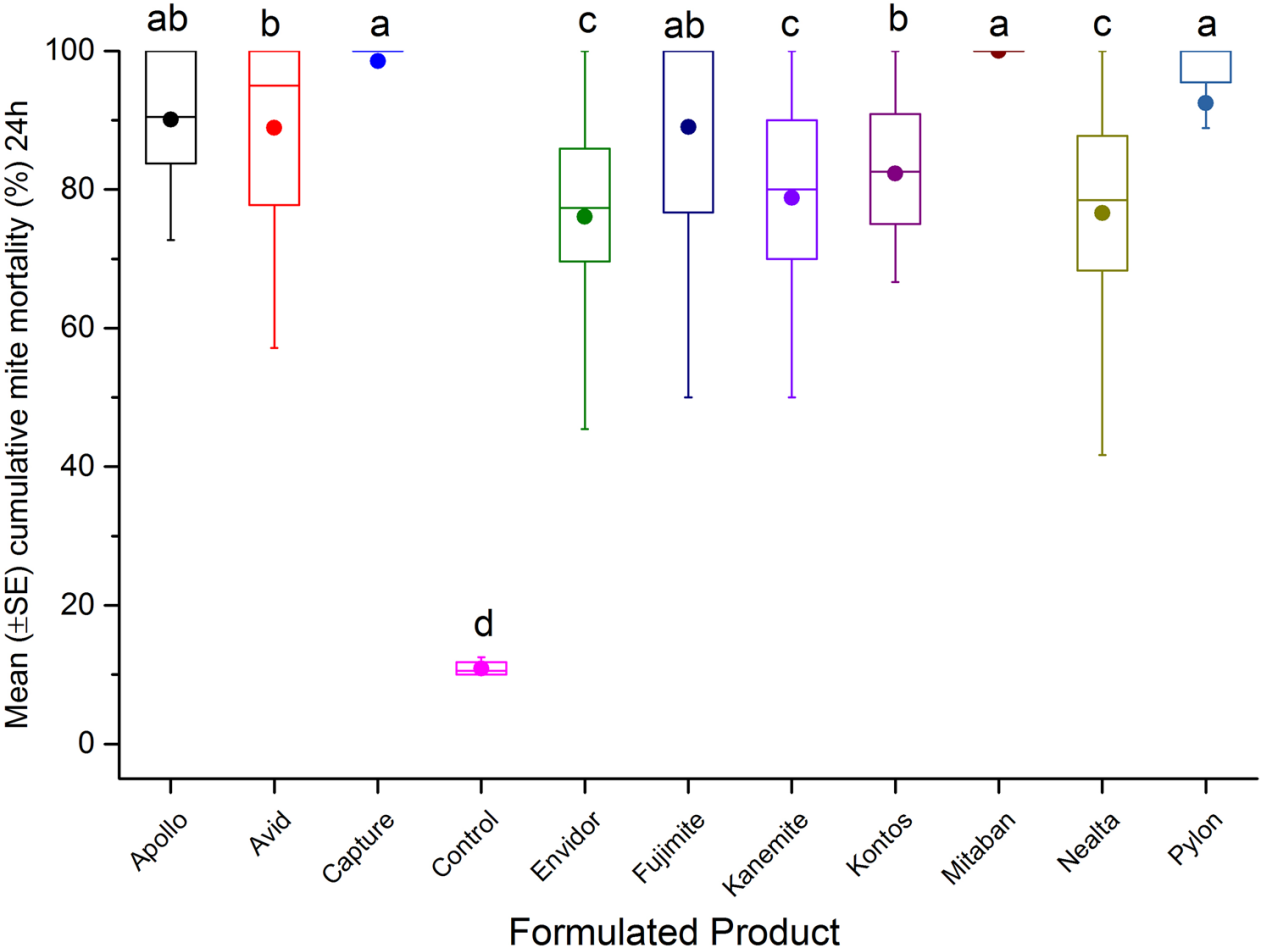
Mean (± SE) cumulative mite mortality (%) during 24 h exposure to different dilutions (0.1 mgL^−1^, 1 mgL^−1^, 10 mgL^−1^, 100 mgL^−1^, 1000 mgL^−1^, or 10,000 mgL^−1^) of tested FPs in acute surface contact toxicity assay using 20 mL borosilicate glass scintillation vials. A group of mites was treated with different dilutions of Mitaban as reference control, or left untreated (control) as negative control. The boxplots present the standard error (length of box), mean (solid circle), median (horizontal line), 5th and 95th percentiles (lower and upper vertical lines). Each box indicates average mortality for replications of each FP (n = 24) or control (n = 4). Means with the same letter among treatments are not significantly different (*p* > 0.05).

In the glass vial test, cumulative mite mortality varied among FPs (n = 10) after the 4 h exposure (F = 12.09; df = 10, 266; p < 0.0001) and 24 h (F = 37.79; df = 10, 266; p < 0.0001) post-treatment periods. Mitaban (74.1 ± 7%) and Capture (64.4 ± 6.4%) had the greatest cumulative mite mortality, compared to other FPs after 4 h exposure (Fig. 3). The highest mortalities were recorded on mites exposed to Mitaban (100 ± 3.1%), Capture (98.5 ± 2.8%), Pylon (92.5 ± 2.8%), Apollo (90.1 ± 3.1%) and Fujimite (89 ± 2.8%) after 24 h (Fig. 4).

#### AIs topical toxicity assay

**Figure 5:**
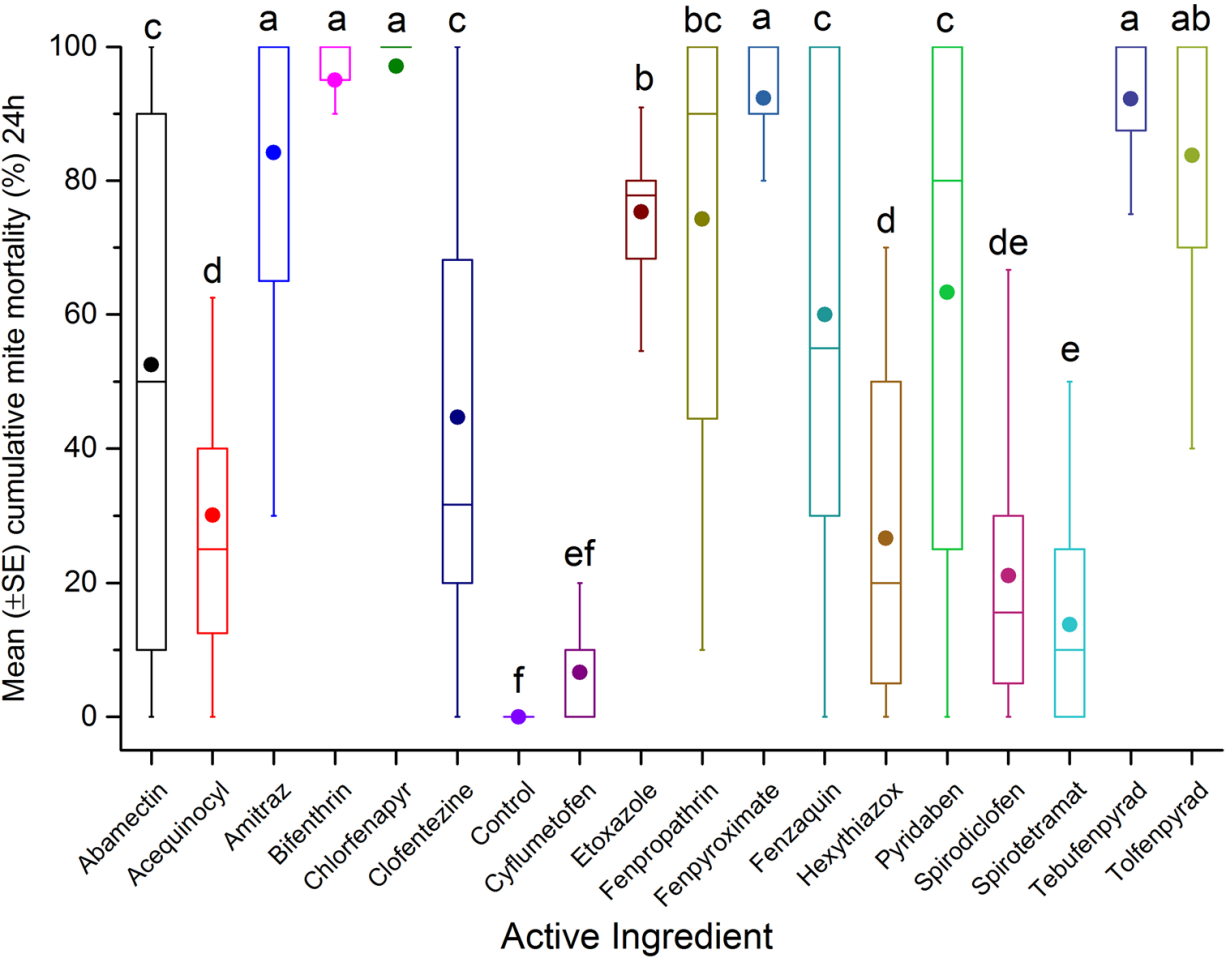
Mean (± SE) cumulative mite mortality (%) during 24 h exposure to different dilutions (0.0000117 µg/mite, 0.000117 µg/mite, 0.00117 µg/mite, 0.0117 µg/mite, 0.117 µg/mite or 1.17 µg/mite) of tested AIs in topical toxicity assay. Each *Varroa* mite was topically treated with 0.15 µL of each AI using micro-applicator. A group of mites was treated with different dilutions of amitraz as reference control, or left untreated (control) as negative control. The boxplots present the standard error (length of box), mean (solid circle), median (horizontal line), 5th and 95th percentiles (lower and upper vertical lines). Each box indicates average mortality for replications of each AI (n = 24) or control (n = 4). Means with the same letter among treatments are not significantly different (*p* > 0.05).

Topical application of the AIs (n = 17) via micro-applicator indicated a higher and non-significant cumulative mite mortality occurred with treatments of: chlorfenapyr (97.1 ± 4.8%), bifenthrin (95 ± 4.8%), fenpyroximate (93 ± 3.4%), tebufenpyrad (92.2 ± 4.8%), and tolfenpyrad (83.8 ± 4.8%) when compared to amitraz (84.2 ± 4.8%). Other AIs presented a significantly lower (≤ 75%) mite mortality rate in comparison to reference control, amitraz (F = 39.88; df = 17, 417; p < 0.0001) (Fig. 5).

### Toxicity assay on honey bees

#### AIs surface contact toxicity assay

Cumulative bee mortality was significantly different among AIs (n = 17) when a group of newly-emerged worker bees were exposed to the contaminated surface of a 60 mL Mason jar for a 24 h period (F = 13.3 ; df = 17, 410; p < 0.0001). Results indicated that fenazaquin, hexythiazox, etoxazole, clofentezine and cyflumetofen were safe on bees and had a similar or lower bee mortality (≤ 28%) compared to amitraz (39%). The other AIs had a high rate of bee mortality (> 40%) in treatment groups (Table 1).

**Table 1:** Mean (± SE) cumulative bee mortality (%) during 24 h exposure to different dilutions of AIs (n = 17) or FPs (n = 10) in acute surface contact (Mason jar) or topical (micro-applicator) toxicity assays.

	Surface contact toxicity assay	Topical toxicity assay
**AIs**		
Abamectin	75.6 ± 7.3^a^	67.1 ± 4.9^ab^
Acequinocyl	40.8 ± 7.3^c^	10.3 ± 4.9^cd^
Amitraz	39 ± 7.3^e^	19 ± 4.9^cd^
Bifenthrin	73.2 ± 7.3^a^	72.3 ± 5^a^
Chlorfenapyr	51.3 ± 7.3^c^	40.9 ± 5^b^
Clofentezine	4.2 ± 7.3^g^	13.4 ± 5.6^cd^
Control (for AIs)	2.5 ± 1.8^g^	1 ± 2^e^
Cyflumetofen	0.4 ± 7.3^g^	4.6 ± 5^de^
Etoxazole	8.7 ± 7.3^fg^	2.8 ± 6.4^de^
Fenazaquin	28.5 ± 7.3^ef^	18.3 ± 6.4^cd^
Fenpropathrin	57.8 ± 7.3^bc^	44.2 ± 6.4^b^
Fenpyroximate	60.9 ± 5.2^bc^	11.9 ± 5.5^cd^
Hexythiazox	21.5 ± 8^f^	1.8 ± 6.4^de^
Pyridaben	53.4 ± 7.3^bc^	30.5 ± 6.4^b^
Spirodiclofen	69.4 ± 7.3^ab^	8.6 ± 4.9^d^
Spirotetramat	61.4 ± 7.3^ab^	9.6 ± 4.9^d^
Tebufenpyrad	81.9 ± 7.3^a^	32.7 ± 6.4^b^
Tolfenpyrad	46.6 ± 7.3^c^	47.3 ± 6.4^b^
**FPs**		
Apollo	7 ± 5.4^e^	7.7 ± 4.8^b^
Avid	98.8 ± 5.4^a^	49.9 ± 4.5^a^
Capture	77.5 ± 5.4^b^	54 ± 4.8^a^
Control (for FPs)	2.5 ± 1.3^e^	1 ± 1.8^b^
Envidor	71.6 ± 5.4^b^	13.4 ± 4.9^b^
Fujimite	32.6 ± 5.4^d^	13.3 ± 4.5^b^
Kanemite	10.9 ± 5.4^de^	7 ± 4.8^b^
Kontos	32.1 ± 5.4^d^	11.8 ± 4.8^b^
Mitaban	89.5 ± 5.4^ab^	6.3 ± 4.9^b^
Nealta	2.1 ± 5.4^e^	37.1 ± 4.8^ab^
Pylon	55.1 ± 5.4^c^	15.2 ± 4.8^b^

Amitraz and Mitaban were tested as reference control for AIs and FPs, respectively.

Means with the same letter in each column among AIs or FPs treatments are not significantly different (*p* > 0.05).

#### FPs surface contact toxicity assay

Among FPs (n = 10), Avid and Mitaban had the highest 24 h cumulative bee mortality (≥ 89%), which was significantly different from all other FPs. Kanemite, Apollo, Nealta and the negative control (no treatment) showed similar bee mortality (≤ 11%) which indicates those treatment groups were safer for bees compared to other FPs (F = 33.63; df = 10, 233; p < 0.0001) (Table 1).

#### AIs topical toxicity assay

For AIs (n = 17) topically treated in the micro-applicator assay, more than 31% of bees were killed during a 24 h-period when treated with bifenthrin, abamectin, tolfenpyrad, fenpropathrin, chlorfenapyr, tebufenpyrad, and pyridaben (F = 19.35; df = 17, 538; p < 0.0001). The rest of the AIs had lower and similar cumulative bee mortality (≤ 19%) compared to amitraz after 24 h using topical application (Table 1).

#### FPs topical toxicity assay

Honey bees topically treated with FPs (n = 10) led to variable mortality during the 24 h post treatment (F = 12.08; df = 10, 348; p < 0.0001). Avid, Nealta and Capture caused significantly higher cumulative bee mortality (≥ 37%) compared to other FPs. However, Pylon, Envidor, Fujimite, Kontos, Apollo, Mitaban and Kanemite had a significantly lower cumulative bee mortality (≤ 15%) and was similar to the control (Table 1).

### Toxicity and selectivity ratios

The mite and bee lethal toxicity values were calculated based on the corrected rates of mortality after 24 h using Abbott’s formula^29^. The greatest toxicity (lower LC_50_) of the AIs to *Varroa* mites after 24 h was observed for etoxazole, spirodiclofen, abamectin, acequinocyl, spirotetramat, clofentezine, pyridaben, hexythiazox, chlorfenapyr and fenpropathrin, when compared to the reference control, amitraz. On the other hand, honey bees had a very low toxicity (higher LC_50_) to only etoxazole when compared to amitraz (Table 2). Comparison of LD_50_ indicated that spirodiclofen and cyflumetofen were both less toxic to *V. destructor* compared to amitraz. Meanwhile, all AIs had similar LD_50_ values for honey bees, compared to the reference control, amitraz (Table 2).

**Table 2:** Lethal concentration fifty (LC_50_) and dose fifty (LD_50_) values for 24 h responses of *V. destructor* and *A. mellifera* to tested AIs (n = 17).

AIs	Mite LC_50_ (mgL^−1^)	Mite LD_50_ (µg/mite)	Bee LC_50_ (mgL^−1^)	Bee LD_50_ (µg/bee)	Toxicity ratio^a^	Selectivity ratio^b^
Abamectin	1.68E−03^C^	9.65E−07^C^	2.79E−01^BCDE^	3.74E−05^CD^	9.69E−05	1.66E+02
Acequinocyl	1.18E−01^C^	3.39E−06^BC^	4.11E−02^BCDE^	2.07E−02^ABC^	6.83E−03	3.47E−01
Amitraz	1.73E+01^B^	1.32E−05^C^	7.02^BCDE^	1.18E−03^ABCD^	1	4.05E−01
Bifenthrin	2.44E+08^A^	2.29E−07^C^	7.64E−04^BCDE^	2.99E−06^CD^	1.41E+07	3.13E−12
Chlorfenapyr	4.07E−02^C^	7.79E−06^C^	2.78E−01^BCDE^	6.95E−05^CD^	2.34E−03	6.84
Clofentezine	6.69E−03^C^	3.48E−07^C^	1.81E+02^C^	3.39E−02^AB^	3.86E−04	2.7E+04
Cyflumetofen	4.31E+06^A^	1.89E−03^AB^	1.08E+02^BC^	7.15E−04^CD^	2.49E+05	2.5E−05
Etoxazole	1.21E−05^C^	7.91E−10^C^	1.38E+03^A^	8.92E−02^AB^	6.98E−07	1.14E+08
Fenazaquin	3.36E+01^B^	2.21E−06^C^	1.93E+01^BCDE^	1.02E−03^CD^	1.94	5.75E−01
Fenpropathrin	8.64E−02^C^	9.3E−06^C^	5.5E−01^BCDE^	3.21E−03^ABCD^	4.98E−03	6.37
Fenpyroximate	3.23E+01^B^	2.98E−06^C^	4.32E−01^BCDE^	1.82E−01^A^	1.86	1.34E−02
Hexythiazox	3.46E−02^C^	1.01E−04^BC^	3.45E−01^BCDE^	1.01E−02^ABC^	1.99E−03	9.98
Pyridaben	2.13E−02^C^	1.13E−05^C^	2.94^BCDE^	3.79E−02^AB^	1.23E−03	1.38E+02
Spirodiclofen	2.73E−04^C^	6.49E−01^A^	9.28E−04^BCDE^	6.27E−03^ABC^	1.58E−05	3.39
Spirotetramat	3.16E−03^C^	4.1E−06^BC^	5.78E−03^BCDE^	5.92E−02^AB^	1.82E−04	1.83
Tebufenpyrad	1.54E+01^BC^	2E−05^BC^	2.72E−03^BCDE^	1.66E−06^CD^	8.9E−01	1.76E−04
Tolfenpyrad	9.55E+01^B^	3.23E−06^C^	5.8^BCDE^	1.61E−09^CD^	5.51	6.07E−02

Amitraz was evaluated as reference control.

Values with overlapping CIs in each column are not considered significantly different as indicated with the same letter (*p* > 0.05).

^a^Toxicity ratio was calculated by dividing the LC_50_ for each AI by LC_50_ for Amitraz.

^b^Selectivity ratio was calculated by dividing the LC_50_ for honey bee by LC_50_ for *Varroa* mite.

Based on the bioassay experiments, etoxazole, clofentezine, pyridaben, hexythiazox, chlorfenapyr, fenpropathrin, spirodiclofen and spirotetramat had a high selectively ratio (> 1), meaning a lower toxicity to bees than to mites. Meanwhile, abamectin, etoxazole, spirodiclofen, spirotetramat, clofentezine, pyridaben, hexythiazox, chlorfenapyr, fenpropathrin, acequinocyl and tebufenpyrad had a higher toxicity ratio (< 1), indicating that these AIs were more toxic to mites than the reference control, amitraz (Table 2). Among FPs evaluated in this study, Nealta and Envidor had a lower mite LC_50_, similar to Mitaban. In the surface contact test, Avid killed bees in all evaluated dilutions; however, Nealta was less hazardous to bees and had higher LC_50_ values than the reference control, Mitaban. The results of topical application indicated lower bee LD_50_ values for Kontos, Capture and Avid compared to Mitaban (Table 3).

**Table 3:** Lethal concentration fifty (LC_50_) and dose fifty (LD_50_) values for 24 h responses of *V. destructor* and *A. mellifera* to tested FPs (n = 10).

FPs	Mite LC_50_ (mgL^−1^)	Bee LC_50_ (mgL^−1^)	Bee LD_50_ (µg/bee)	Toxicity ratio^a^	Selectivity ratio^b^
Apollo	1.89E−01^C^	1.7E+02^A^	7.85E−03^ABC^	5.12E+01	9E+02
Avid	3.01E+01^B^	–	3.68E−04^C^	8.14E+03	–
Capture	3.6E+02^B^	1.3E−01^C^	2.06E−04^C^	9.73E+04	3.62E−04
Envidor	1.42E−04^D^	6.35E−03^C^	6.57E−04^BC^	3.84E−02	4.47E+01
Fujimite	9.86E+01^B^	7.31^BC^	3.04E−01^A^	2.66E+04	7.42E−02
Kanemite	2.42E−02^C^	3.88^BC^	2.19E−02^ABC^	6.54	1.61E+02
Kontos	9.88E+06^A^	3.18^BC^	1.24E−05^C^	2.67E+09	3.22E−07
Mitaban	3.7E−03^CD^*	5.33E−02^C^	7.1E−02^AB^	1	1.44E+01
Nealta	3.81E−05^D^	1.4E+02^B^	3.82E−04^BC^	1.03E−02	3.68E+06
Pylon	1.85E+03^B^	1.01^C^	1.58E−01^A^	5E+05	5.47E−04

Mitaban was evaluated as reference control.

Values with overlapping CIs in each column are not considered significantly different as indicated with the same letter (*p* > 0.05).

^a^Toxicity ratio was calculated by dividing the LC_50_ for each AI by LC_50_ for Mitaban.

^b^Selectivity ratio was calculated by dividing the LC_50_ for honey bee by LC_50_ for *Varroa* mite.

*The mite LC_50_ value for Mitaban was determined using serial dilutions of 0.0001–100 mgL^−1^.

Overall, laboratory bioassay results demonstrated potential Varroacide activity for etoxazole, fenazaquin, fenpyroximate, spirodiclofen and spirotetramat all having modes of action different from amitraz (formamidine), and with low adverse affects on bees. However, among these compounds, etoxazole and fenazaquin were the most promising candidates for a new Varroacide.

## Discussion

Few laboratory or field toxicity investigations have targeted testing the efficacy and safety of commercial miticides on *V. destructor* and *A. mellifera*^24–26^. Many of the miticides evaluated in this study (e.g. abamectin, chlorfenapyr, cyflumetofen, etoxazole, fenpropathrin, fenazaquin, hexythiazox, pyridaben, spirodiclofen, spirotetramat, and tolfenpyrad) were evaluated globally for the first time on honey bees and *Varroa* mites. Results suggest that among the 16 AIs evaluated in the laboratory trials etoxazole, fenazaquin, fenpyroximate, spirodiclofen and spirotetramat with different modes of action to those currently registered as Varroacides, show the potential to control *Varroa* mite. Among the AIs, etoxazole and fenazaquin had the highest mite control efficacy with minimal effects on honey bees. These AIs should be subjected to further testing at the honey bee colony level to determine the effective dose, optimal application method and their effects on colony performance and *A. mellifera* fitness.

Beekeepers use a variety of miticides to control *Varroa* mite populations including Apivar (amitraz). Apivar has been used in Canada for more than a decade and remains an effective option for beekeepers. This product has been registered in the USA since 2013, and recently Rinkevich^12^ reported resistance of *Varroa* mites to amitraz in Louisiana, New York, and South Dakota beekeeping operations. However, in this study, amitraz efficacy was > 90% at dilution of > 10 mgL^−1^ indicating that amitraz remains an effective product for the control of *V. destructor* in the tested mite population. Despite the wide use of amitraz as a Varroacide, globally, few studies examined the toxicity of amitraz on mites and bees. Santiago et al*.*^30^, for instance, evaluated the lethal toxicity values of amitraz for *Varroa* mite as LC_50_ = 0.23 mgL^−1^ and LD_50_ = 0.0000017 µg/mite; and for honey bee as LC_50_ = 1.64 mgL^−1^ and LD_50_ = 2.55 µg/bee. According Abed et al*.*^31^, amitraz LD_50_ was 0.00000216 µg/mite for *Varroa* mites. Maggi et al*.*^28^ exposed *Varroa* mites to amitraz in Petri dishes and reported a high toxicity (LC_50_ = 0.1 μg/dish) to mites. However, Elzen et al*.*^32^ found a higher amitraz LC_50_ value (16.35 μg/vial) for mites in a glass vial assay. Our studies determined the amitraz LD_50_ and LC_50_ values in the tested mite population to be 0.0000132 µg/mite and 17.34 mgL^−1^, when using the glass vial and micro-applicator methods, respectively. The difference between studies could be due to any of the following: variabilities in mite populations, history of exposure to amitraz, duration of bioassay, methods and statistical analyses.

The surface contact application (glass vial assay) showed a rapid and simple method to screen new compounds having Varroacidal properties. Consistent with other studies^20,22,23^, this method can also be adopted for assessment of *Varroa* mite populations resistance to miticides. Frequent monitoring for *Varroa* resistance to synthetic Varroacides are important for honey bee colony health and the long-term sustainability of the beekeeping industry in Canada. In the past, mite resistance was evaluated using a variety of methods, with mite susceptibility to chemicals varying depending on the region. Kamler et al*.*^21^ developed a polypropylene vial (instead of glass vial) bioassay and documented a higher toxicity for amitraz in the resistant (LC_50_ = 0.00802 mgL^−1^) compared to the susceptible (LC_50_ = 0.25104 mgL^−1^) mite populations during a 24 h-period. The studies^30,33^ conducted in Mexico on susceptible mites estimated LC_50_ for amitraz to be 0.23–0.526 mgL^−1^ using the Petri dish, topical and spraying methods. In our study, mites were collected from high-*Varroa* infested colonies with no reported resistance to amitraz. The bioassay established an LC_50_ reference point for amitraz of 17.34 mgL^−1^, for susceptible *Varroa* mite populations tested in this study.

Other research has focused on exposing honey bee workers to pesticides using feeding^34^, surface contact in the Petri dishes^35^ and topical^36,37^ methods. In this study, newly-emerged worker bees were exposed to chemicals by coating the inside of 60 mL glass Mason jar. When specifically looking at amitraz and the formulated product Mitaban, the bioassay confirmed a significantly higher bee mortality when bees were exposed to higher dilutions of both compounds. For example, when the inner surface of jars (67.75 cm^−2^) were coated with 1000 mgL^−1^ amitraz, bees were exposed to 0.0074 mgcm^−2^ (approximately 0.074 mg/bee) of amitraz, which caused 100% mortality. This result is not surprising because when *Varroa*-infested colonies are treated with Apivar, bees are exposed 5 mgcm^−2^ (approximately 0.00042 mg/bee) of the AI (amitraz) over a 6–8 week treatment period. To explain high bee mortality in the Mason jar experiment, bees were exposed to a larger quantity of amitraz for a shorter period of time and in a confined space, unlike a colony environment.

Other evaluated AIs in the surface contact method had different adverse effects on bees when compared to amitraz. Johnson et al*.*^37^ reported bees having a higher toxicity to amitraz compared to bees exposed to fenpyroximate, tau-fluvalinate, coumaphos and thymol. The tolerance of bees towards some miticides such as tau-fluvalinate, coumaphos and fenpyroximate appear to be associated to P450s detoxification activities^37^. For honey bees, the mode of action of amitraz is unknown. Dahlgren et al*.*^25^ determined that for amitraz, honey bee LD_50_ value was 0.00267%, when using the topical application (1 µL/bee) on the thorax, with this value being higher than the our study where the value was determines to be 0.00084% (= 0.00045 µg/bee). They also found that amitraz (LD_50_ = 21.8 µgg^−1^) was more toxic to queens than fenpyroximate (LD_50_ > 1620 µgg^−1^)^25^. Queens, on the other hand, had a higher tolerance to amitraz and fenpyroximate than workers did. Amitraz was also toxic to bees when treated orally (LD_50_ = 7.082 µL/bee) for 24 h^38^, with higher mortality rate (> 60%) at dilutions greater than 1000 mgL^−1^^38,39^. In our study, oral toxicity was not evaluated, but cumulative bee mortality was less than 41% when bees were in contact with amitraz at ≤ 100 mgL^−1^ in the glass Mason jars.

Yashima discovered etoxazole from the oxazoline chemical class in the 1980s^40^. This AI was commercialized in 1998^41^ for the control of immature mites, aphids, moths and other phytophagous insects in crop. Our trials were the first to investigate the effect of etoxazole on *V. destructor* and *A. mellifera*. It was found that etoxazole had a higher toxicity (LC_50_ = 0.0000121 mgL^−1^) to mites compared to amitraz and was safe for use on honey bee. The vial test supported the result, with an acceptable efficacy (70%) of mite control when using etoxazole, compared to amitraz (77%). Meanwhile, the mortality rate was < 9% when bees were exposed to 0.1–10,000 mgL^−1^ of etoxazole in Mason jars. Overall, etoxazole was effective in the bioassay tests under laboratory conditions, and it is recommended that future evaluation take place under field conditions.

Fenazaquin from the chemical class quinazoline was introduced in 1988 by Dow AgroSciences^42^. Quinazoline products were reported to have minimal impact on many beneficial insects and mites^43^. On target species, fenazaquin is known to inhibit the mitochondrial electron transport chain and has been used against spider mites. For the Asian honey bee, *Apis cerana* F., a 60% mortality was observed thirty minutes after sunflower leaves were treated with fenazaquin, but toxicity dropped to 9.6% when exposure occurred one day after treatment. The toxicity (LD_50_) of fenazaquin to *A. cerana* was 1.5–508 µg/bee^44^. Our study is the first to evaluate the effects of fenazaquin on *A. mellifera* and *V. destructor.* The surface contact toxicity results revealed that fenazaquin had the potential to kill 76% and 92% of *Varroa* mites, after a 4 h exposure and 24 h post-treatment, respectively. Fenazaquin had no negative effects on bees at lower dilutions (< 100 mgL^−1^) which also resulted in low rates of bee mortality (0–12%) similar to that of amitraz. Fenazaquin toxicity (LC_50_ = 33.62 mgL^−1^) on mites was similar to amitraz. Laboratory bioassays indicated that fenazaquin was less harmful to bees compared to Mitaban, but only in the surface contact test. On the other hand, fenazaquin along with tebufenpyrad and fenpyroximate resulted in rapid mite kill in a short period after 4 h exposure in the glass vial test. The results suggest that fenazaquin could be further evaluated under field conditions as a potential Varroacide.

This study also evaluated a group of miticides belonging to the pyrazole class of chemical (Supplementary Table S1). These chemicals target mitochondrial complex I, with low to moderate effects on beneficial insects and predator mites. Miticides examined in this study were: tebufenpyrad, tolfenpyrad and fenpyroximate. Our results revealed that tebufenpyrad may be an effective miticide to control *V. destructor*, due to a high cumulative mite mortality (73%), which was similar to fenazaquin (76%) and fenpyroximate (63%), but significantly higher than amitraz (55%), when tested 4 h exposure in surface contact test. Ali et al*.*^24^ exposed honey bee colonies to tebufenpyrad and found that *Varroa* mite abundance dropped by 77%. At the same time, the use of tebufenpyrad resulted in a reduced brood area, adult population, and an increase incidence of queen failure. With findings consistent with Ali et al*.*^24^, we found unacceptable bee mortality in jars (82%) and in topical (33%) treatment methods. Despite finding high rates of bee mortality, further study may identify an effective dose for control of mites, while remaining safe for use on honey bees.

In the pyrazole group, fenpyroximate was commercialized in 1991 by Nihon Noyaku^45^ and introduced as Hivastan in the USA in 2007 for the control *V. destructor*^46^. This product was not registered for use as a Varroacide in Canada. Hivastan was formulated and applied to colonies in a patty containing 0.3% of the AI. To date, there is minimal evidence for its efficacy at controlling *Varroa* mites, or its adverse effects on bees. Hivastan was only used in the USA (2008–2010)^46^, and due to high bee kill following an application of the registered formulation, the registrant pulled the registration with the Environmental Protection Agency (EPA). Further investigations have reported that fenpyroximate affects *A. mellifera*^25,47^. For example, fenpyroximate increased aggression by 31% in treated bees^47^ and caused fewer recapturing of drones two weeks post-treatment^37^. Our result showed a lower level of toxicity (LD_50_ = 0.023%) for fenpyroximate compared to a previous report (0.003%)^25^. Furthermore, our results also showed that newly-emerged worker bees tolerated fenpyroximate at lower doses, while cumulative mite mortality was > 60%, when exposed to < 10 mgL^−1^ of AI. It was also observed that significantly higher rates of cumulative mite mortality for fenpyroximate occurred during the 4 h (63%) or 24 h (92%) compared to the reference control (55% and 76%, respectively). Currently, no evidence is available documenting *Varroa* mite resistance to fenpyroximate. However, Kim et al*.*^48^ identified fenpyroximate resistance in two-spotted spider mite (*T. urticae*). Although Hivastan is no longer available as a tool for controlling *Varroa* mites, it is recommended that this product be re-investigated to determine an effective dose against mites, at a level safe for bees under field conditions.

Tetronic acids are a group of miticides including spirodiclofen and spirotetramat that inhibit lipid biosynthesis. They are used to control a number of phytophagous mites. Spirodiclofen was commercialized by Bayer CropScience in 2002. Only a few studies looked at the influence of tetronic acid on *A. mellifera* and *V. destructor.* In a trial, the product Envidor (spirodiclofen) was found to have an LD_50_ of > 200 µg/bee with negative effects on brood development^49^. In our study, the LD_50_ for newly emerged worker bees exposed to Envidor was 0.00066 µg/bee. Envidor had a high bee mortality rate (> 70%) in the glass jar test, similar to bees treated with its AI, spirodiclofen. However, Envidor was safe on bees in topical application (13% mortality). No peer reviewed studies on the toxicity of spirodiclofen to bees or mites were found, but Vandervalk^22^ showed that another tetronic acid, Forbid (spiromesifen 45.20%), effectively controlled *Varroa* mite at higher dilution (10%) in the glass vial test. Despite the high bee death rate for newly-emerged bees exposed to spirodiclofen, our results indicated a 76–84% mortality in *V. destructor* for the AI or FP applications. It is recommended more research on spirodiclofen be conducted to determine an effective dose to kill *Varroa* mites, and an application method to minimize the negative effects on honey bees.

Spirotetramat is a systemic miticide used against many crop pests^50^. The contact toxicity on *A. c. indica* when spirotetramat was used in the field at 40, 60 and 75 gha^−1^ resulted in 10%, 16.67% and 30% mortality, respectively, in honey bee populations^51^. Nevertheless, Maus^50^ concluded that when spirotetramat was applied orally (107.3 μg/bee) and via contact (> 100 μg/bee), it remained safe for use on *A. mellifera*. In our study, when adult bees were exposed to either the AI or FP by topical application, spirotetramat did not result in a significant increase in the 24 h mortality rate (< 12%). However, a high mortality (61%) was observed when bees were exposed to compounds using the surface contact method. When mites were exposed to spirotetramat, *V. destructor* mortality was high (84%) and similar to the reference control (77%). Our laboratory results suggest that spirotetramat should be examined as a potential miticide; however, determining an effective dose for controlling *Varroa* mites in the field, and reducing the negative effect of spirotetramat on honey bees at the colony level, are required.

The standard approach to evaluating the acute toxicity of miticides on *Varroa* mites or honey bee is to determine the LD_50_ or LC_50_ values and the mortality rates a given active ingredient on animals using bioassay techniques. In this project, we described bioassay procedures for screening potential compounds for *Varroa* control and bee safety. The assays included testing compounds under laboratory conditions for mite and bee mortality using the surface contact (glass vial or glass Mason jar) and topical application (micro-applicator) methods. In the search for alternative synthetic product options to control *Varroa* mites, the toxicity and mortality values of 17 AIs, 12 of which were evaluated for the first time on *Varroa* mites and/or honey bees, were determined. Overall, abamectin, acequinocyl, bifenthrin, chlorfenapyr, clofentezine, cyflumetofen, fenpropathrin, hexythiazox, pyridaben, and tolfenpyrad either had limited efficacy against mites, or had high bee mortality. These AIs were not considered for future laboratory field studies as potential *Varroa* control product.

In conclusion, this research has established initial bioassay procedures for screening potential compounds against *Varroa* mites. Our results suggest that AIs such as fenazaquin and etoxazole succeeded in controlling *Varroa* mites under laboratory conditions and are relatively safe for use on honey bees. Fenpyroximate, spirodiclofen, and spirotetramat had relatively high efficiency in mite control, but further investigations are required to mitigate negative effects on *A. mellifera*. Although laboratory screening of these compounds has proved to be effective against *Varroa* mites, this is only an initial testing phase, and use of these compounds in colonies is not legal or advised. Further research is required to determine the compound’s effects on: colony performance, colony fitness, sub-lethal effects of miticide residues on queens, drones and brood, operator safety, residues in honey and wax, and their safety for human consumption. The next step in the development of an effective miticide would be to conduct more extensive research to assess these compounds under field conditions on full size colonies. We also recommend screening more AIs with different modes of action, and the use of synergists in the bioassay trials, to enhance the efficacy of miticides. Once effective and safe compounds are identified and confirmed at the colony level, the results would support future development of a Varroacide and beekeepers with an additional management tool, thereby adding significant economic value to the beekeeping and agricultural industries.

## Methods

The laboratory trials were conducted at the Crop Diversification Center North (CDCN), Edmonton, Alberta, Canada (53.54° N, 113.49° W) in summer 2016–2018. All experimental bee colonies used in this bioassay study were European honey bee (*A. mellifera*) colonies that were managed under the same management practices at CDCN to reduce variabilities among tested *Varroa* mite and honey bee populations. The cumulative mortality rates of *V. destructor* and *A. mellifera* mortality, and the toxicity of 17 Als (abamectin, acequinocyl, amitraz, bifenthrin, chlorfenapyr, clofentezine, cyflumetofen, etoxazole, fenpropathrin, fenpyroximate, fenazaquin, hexythiazox, pyridaben, spirodiclofen, spirotetramat, tebufenpyrad, and tolfenpyrad), and 10 FPs (Apollo, Avid, Capture, Envidor, Fujimite, Kanemite, Kontos, Mitaban, Nealta and Pylon) were tested using surface contact (glass vials and Mason jars) and topical (micro-applicator) methods. In all bioassay tests, if the average of the mite or bee mortality in the negative controls were > 15%^52^, the samples were discarded and the test repeated.

### Chemical preparations

The following commercial FPs were obtained from Terralink, BC, Canada: Avid (abamectin, 1.98%), Kontos (spirotetramat, 22.4%), Capture (bifenthrin, 24%), Pylon (chlorfenapyr, 24%), Fujimite (fenpyroximate, 5%), and Envidor (spirodiclofen, 24%). Other FPs were Nealta (cyflumetofen, 20%, Bartlett, ON, Canada) and Mitaban (amitraz, 19.9%, Zoetis, USA). All AIs were obtained from Sigma-Aldrich, ON, Canada, except cyflumetofen (Cedarlane, NC, USA) (Table 1). Acetone (Fisherbrand, Fisher Scientific, Canada) and water were used as solvents for the AIs and FPs, respectively. Acetonitrile (Sigma-Aldrich, USA) was used as solvent for clofentezine. Fresh stock solutions for each FP (100,000 mgL^−1^ = 10%) or AI (10,000 mgL^−1^ = 1%) were made in 15 mL polypropylene centrifuge tubes (Fisherbrand, Fisher Scientific, Canada) using water (density 1 gmL^−1^), acetone (density 784 gmL^−1^) or acetonitrile (density 786 gmL^−1^), respectively. The stock solutions were agitated on a vortex mixer (VWR, USA) for a period of 2–3 min, and prepared for same day use. All interactions with chemicals were done under the fume hood with operators wearing a full-face respirator mask (6900, 3M, USA) including filters (60923, 3M, USA) and other PPE. In laboratory trials, reference controls (Mitaban for FPs and amitraz for AIs), death controls (dimethoate^53^ 0.000033 mgL^−1^ and amitraz 100,000 mgL^−1^ for bees and mites, respectively), solvent controls (water, acetone and acetonitrile), and negative control (no treatment) served as control treatments.

### Mite collection, newly-emerged bees and brood preparation

Two sets of experimental honey bee colonies were provided for this investigation. The first group had high *Varroa* mite infestation levels (> 3%), and were placed in a separate yard without any Varroacide treatment. Every colony in this yard received two drone frames in early July to increase *Varroa* mite populations for the experiment. The mites from the highly infested colonies were used in the vial experiment and resistance test. The second set of colonies was kept healthy with no or low mite levels (< 1%) in another yard at CDCN. These colonies were used to extract purple-eyed pupae for feeding during the vial test and to supply newly emerged bees for the Mason jar test. The initial arithmetic mean abundance^54^ of *Varroa* mites in colonies was determined using the alcohol wash method. In this method, a sample of 300 bees was collected in 500 mL plastic containers filled with 250 mL of ethanol (70%), from brood frames sourced from CDCN colonies with high mite infestation (> 3%). The *Varroa*-infested bee samples were agitated on an orbital shaker at 300 rpm for 10 min. The number of mites were counted by placing the bee samples in a sieve over a white container. Bees were washed using tap water to dislodge the mites. Mites that fell into the container were counted, and bees were individually counted to calculate mite infestation level. The evaluation of *Varroa* mite resistance to Apivar took place on the same highly infested colonies before mite collection. An adapted version of the Pettis method^55^ was used to ensure that mites in the experiments were susceptible to amitraz and could be used in the bioassay as a reference control. Using the Pettis method^55^, a group of 150 worker bees were exposed to a piece of Apivar strip (2.54 × 6.35 cm) that was stapled on a piece of cardboard and placed in a 500 mL Mason jar (Bernardin, Canada). The jars with bees were incubated at 25 °C and 50–60% RH in the dark, and the number of dead and live mites were counted 24 h post-exposure. To collect live *Varroa* mites for the vial test, 300–500 g *Varroa*-infested bees from the highly infested colonies were transferred into a Plexiglass shaker basket (8.5 × 14 × 28.5 cm) with a metal mesh bottom (12.5 × 27.5 cm). The Plexiglass basket rested inside a Rubbermaid container (12 × 20 × 35 cm) with a fitted lid imbedded with one inlet CO_2_ valve. The container and bees were agitated at 300 rpm on a mechanical shaker while being exposed to CO_2_ (5 Lmin^−1^) for 5 min and another 5 min without CO_2_. Mites that fell through the mesh onto the bottom of the container were collected with a fine-tipped paintbrush and placed in Petri dishes lined with a moist paper towel to prevent desiccation of the live mites^56^. Capped brood frames were taken from healthy colonies (< 1% mite infestation) to provide fresh pupae to sustain mites during the vial experiment. Purple-eyed pupae were carefully removed from cells using forceps and placed on a damp paper towel in a Petri dish with a cover and incubated at 33 ± 1 °C and 60 ± 5% RH in the dark until required for experiment. In order to collect newly-emerged bees for the Mason jar test, frames of sealed brood free of worker bees were removed from healthy colonies and individually confined in wooden brood emergence cages (50 × 26 × 7 cm). Each emergence cage had a screen on both sides for ventilation and was kept in an incubator at 33 ± 1 °C and 60 ± 5% RH in the dark until bees emerged. Once emerged, the bees were collected in a plastic Rubbermaid container and exposed to CO_2_ for handling.

### Acute surface contact toxicity assay of miticides on Varroa mites (Vial test)

Subsequent serial dilutions of 10,000 mgL^−1^, 1000 mgL^−1^, 100 mgL^−1^, 10 mgL^−1^, 1 mgL^−1^ and 0.1 mgL^−1^ were prepared in 15 mL polypropylene centrifuge tubes for AIs (n = 17) or FPs (n = 10). Borosilicate scintillation glass vials (20 mL, Cole-Palmer, QC, Canada) were treated with 0.5 mL of each dilution using a pipette, and each dilution had four replicates per compound. The glass vials were rotated horizontally on a hot dog roller (Addcraft, Hicksville, NY, USA) under a fume hood at room temperature for 2–3 h until solvents completely evaporated and the residue of the compounds homogenously coated the inner side-surface of vials (40.36 cm^2^). To do this, the hot dog roller functioned normally without any modifications, except the heating element was turned off. Eight to ten live and freshly collected mites were placed into the treated vials using a new-labelled brush (Regular size Ultrabrush, Microbrash, WI, USA). To avoid cross-contamination, a new brush was used for each dilution of chemicals. Prepared vials with mites were incubated (She Lab, Sheldon Inc., OR, USA) at 33 ± 1 °C and 60 ± 5% RH in the dark. The temperature (°C) and relative humidity (%) in incubator were monitored using HOBO (Onset Computer Corporation, MA, USA) data loggers. Mite mortality was counted after 4 h of exposure and the surviving mites were transferred into a clean polypropylene 2 mL centrifuge tube (Fisherbrand, Fisher Scientific, Canada) with holes poked on top for ventilation. Each tube contained one honey bee purple-eyed pupa for feeding. Tubes were incubated at 33 ± 1 °C and 60 ± 5% RH in the dark for an additional 20 h. Mite mortality was determined 24 h post-treatment by transferring them into a Petri dish. Mortality was assessed by gently probing mites using a fine-tipped paint brush under a magnifying glass to detect subtle limb movement. Mites that were completely motionless (i.e. lack of appendage movement), while probing were considered dead. Control treatments included three solvents (acetone, acetonitrile and water), two reference controls (amitraz and Mitaban) and a negative control (no treatment).

### Acute topical toxicity assay of miticides on *Varroa* mites (micro-applicator test)

A digital Nano-pump (KDS310,Cole-Parmeer, IL, USA) fitted with a 10 µL micro-syringe (701RN, Hamilton Company, NV, USA) and small hub needle ( 22 s gauge, 0.75 in, point style 3, Hamilton, Company, NV, USA) was used to apply candidate compounds onto mites. For each compound of AIs (n = 17), 8 to 10 live and freshly collected mites were topically treated with 0.15 µL on the dorsal shield to receive 0.0000117 µg/mite, 0.000117 µg/mite, 0.00117 µg/mite, 0.0117 µg/mite, 0.117 µg/mite, or 1.17 µg/mite, with four replicates for each dilution. Treated mites were placed directly into 2 mL polypropylene centrifuge tubes containing one purple-eyed pupa for feeding using a new-labelled micro brush for each dilution. Tubes were incubated at 33 ± 1 °C and 60 ± 5% RH in the dark for 24 h. Mite mortality was assessed at 24 h post-treatment. Control treatments included three solvents (acetone, acetonitrile and water), a positive control (amitraz) and a negative control (no treatment).

### Acute surface contact toxicity assay of miticides on honey bees (Mason jar test)

The acute surface contact toxicity of compounds for honey bees were evaluated using Mason jars (60 mL, Uline, AB, Canada). Using a pipette, mason jars were treated with 0.5 mL of each chemical dilution for the AIs (n = 17) or FPs (n = 10) (0.1 mgL^−1^, 1 mgL^−1^, 10 mgL^−1^, 100 mgL^−1^, 1000 mgL^−1^ or 10,000 mgL^−1^) with four replicates per dilution per compound. The Mason jars were horizontally rotated on a hot dog roller, like the glass vials, under a fume hood at room temperature for 2–3 h until solvents completely evaporated and the residue of compounds homogenously to coated the inside surface of the jars (67.75 cm^2^). To do this, the hot dog roller functioned normally without any modifications, except the heating element was turned off. One sugar cube was glued to the inside-bottom of the jar before 10 newly emerged bees were added. The top of the jar was covered using a fine mesh screen (16 mesh, Easy Screen, RCR International Inc., Canada) that was secured using an elastic band. The bees were incubated at 33 ± 1 °C and 60 ± 5% RH in the dark for 24 h. After 24 h exposure treatment, bee mortality was assessed. Bees were determined to be dead if they were completely motionless (i.e. no body or appendage movement), when the jar was slightly agitated. Control treatments included three solvents (acetone, acetonitrile and water), two positive control (amitraz and Mitaban) and a negative control (no treatment).

### Acute topical toxicity assay of miticides on honey bees (micro-applicator test)

Using the same methodology for topically treating *Varroa* mites, the micro-applicator was fitted with a 25 µL micro-syringe (702RN) and small hub needle (22 s gauge, 0.75 in, point style 3) to apply each candidate compound. For dilutions of AIs (n = 17) or FPs (n = 10) (0.000078 µg/bee, 0.00078 µg/bee, 0.0078 µg/bee, 0.078 µg/bee, 0.78 µg/bee, or 7.8 µg/bee), a group of 20 newly emerged worker bees were anesthetized by exposure to CO_2_ and individual bees were topically treated with 1 µL of the each compound dilution applied to the thorax. Each treatment had four replicates. Once treated, the bees were placed in 125 mL plastic containers (Plastipak Industries Inc., SK, Canada) and fed one sugar cube. Holes were made on the body of container for air ventilation. The bees were incubated at 33 ± 1 °C and 60 ± 5% RH in the dark for 24 h. Bee mortality was assessed 24 h post exposure. Bees were determined to be dead if they were completely motionless (i.e. no body or appendage movement), when the container was slightly agitated. Control treatments included three solvents (acetone, acetonitrile and water), two positive controls (amitraz and Mitaban) and a negative control (no treatment).

### Statistical analysis

The variables for cumulative mite and bee mortality rate (%) were analyzed using a mixed model ANOVA (PROC MIXED)^57^ in which compounds were treated as main plots, dilutions as sub plots and replicates as random effects. The cumulative mortality was estimated as a proportion of dead mites or dead bees in all replicates of dilutions in each compound. Normality of the data was analyzed using Shapiro–Wilk test (PROC UNIVARIATE)^57^. Proportion for cumulative mortality rates in all trials were arcsine transformed prior to analyses^58^. Data are presented as untransformed means. Where significant interactions occurred, differences among treatment means were compared using the Bonferroni correction. The lethal concentration fifty (LC_50_) and lethal dose fifty (LD_50_) of compounds to *V. destructor* and *A. mellifera* at 24 h were evaluated using PROC PROBIT^57^. The percentage of mite mortality was corrected using Abbott’s correction^29^ as corrected mortality (%) = (% mortality in treatment − % mortality in control)/(100 − % mortality in control). The toxicity ratios (AI LC_50_/amitraz LC_50_) and selectivity ratios (*A. mellifera* LC_50_/*V. destructor* LC_50_) at 24 h post exposure were determined. If confidence intervals (CI) overlapped, the difference between toxicity values were evaluated as not statistically significant.

## Supplementary Information

Supplementary Information.
