# Supplementary material for: Evaluation of potential miticide toxicity to Varroa destructor and honey bees, Apis mellifera, under laboratory conditions
Source: Sci Rep. 2020 Dec 9;10:21529. doi: 10.1038/s41598-020-78561-2 (PMC7726572; doi:10.1038/s41598-020-78561-2)
Supplement: Supplementary file 1 — Supplementary Information. [file 41598_2020_78561_MOESM1_ESM.pdf]

Evaluation of potential miticide toxicity to *Varroa destructor* and honey bees*, Apis mellifera,* under laboratory conditions

Rassol Bahreini[1*], Medhat Nasr[1], Cassandra Docherty[1], Olivia de Herdt[1], Samantha Muirhead[1] & David Feindel[1]

[1]Plant and Bee Health Surveillance Section, Alberta Agriculture and Forestry, 17507 Fort Road NW, Edmonton, Alberta, T5Y 6H3, Canada

[*]Corresponding: rassol.bahreini@gmail.com

Classification: Public

Supplementary Table S1: List of FPs and their associated AIs that were evaluated in this study from different chemical classes with different modes of action.

FP	AI	Guarantee (%)	Class	Mode of action
Apollo	Clofentezine	50	Tetrazines	Growth inhibitor
Kanemite	Acequinocyl	15.8	Quinolines	Inhibits the respiration of mitochondria
Envidor	Spirodiclofen	24	Tetronic acids	Inhibitor of lipid biosynthesis
Mitaban	Amitraz	19.9	Formamidines	Octopamine receptor agonist
Avid	Abamectin	1.98	Avermectins	GABA agonist
Kontos	Spirotetramat	22.4	Tetronic acids	Inhibitor of lipid biosynthesis
Capture	Bifenthrin	24	Pyrethroids	Effect on voltage-sensitive ion channels
Pylon	Chlorfenapyr	24	Pyrroles	Effect on oxidative phosphorylation
Fujimite	Fenpyroximate	5	Pyrazoles	Inhibitor of ATP synthase
Nealta	Cyflumetofen	20	Benzoylacetonitriles	Inhibitor of mitochondria complex II
-	Hexythiazox	-	Thiazolidinones	Growth inhibitor
-	Etoxazole	-	Oxazolines	Growth inhibitor
-	Fenazaquin	-	Quinazoline	Inhibitor of lipid biosynthesis
-	Pyridaben	-	Organochlorines	Hyperstimulation of nerve transmission
-	Fenpropathrin	-	Pyrethroids	Effect on voltage-sensitive ion channels
-	Tebufenpyrad	-	Pyrazoles	Inhibitor of ATP synthase
-	Tolfenpyrad	-	Pyrazoles	Inhibitor of ATP synthase

Classification: Public

Supplementary Table S2: Mean (±SE) mite and bee mortality (%) during 24 h exposure to different solvents in acute surface contact (glass vial or Mason jar) or topical (micro-applicator) toxicity assays.

Solvent	Dilution (%)	Mite/bee	Toxicity assay	Mean mortality ± SE (%)
Acetone	99.90	Mite	Surface contact	10.6±4.4
Acetone	99. 90	Mite	Topical	2.1±4.1
Acetone	99. 90	Bee	Surface contact	3.6±1.6
Acetone	99. 90	Bee	Topical	4.4±2.1
Acetonitrile	99. 80	Mite	Surface contact	35.5±4.4
Acetonitrile	99.80	Mite	Topical	15±4.1
Acetonitrile	99.80	Bee	Surface contact	5.2±1.5
Acetonitrile	99.80	Bee	Topical	5.6±1.8
Water	-	Mite	Surface contact	2.8±4.4
Water	-	Bee	Surface contact	1±1.5
Water	-	Bee	Topical	0

Classification: Public

Supplementary Table S3: Mean (±SE) mite and bee mortality (%) during 24 h exposure to different dilutions of amitraz and control in acute surface contact (glass vial or Mason jar) or topical (micro-applicator) toxicity assays. Means with the same letter among dilations for each assay are not significantly different (*p*>0.05).

Product	Toxicity assay	Mite/bee	Dilution	Mean mortality ± SE (%)
Amitraz	Surface contact	Mite	0 (mgL^{-1})	5±4.8[d]
			0.1 (mgL^{-1})	49.2±4.8[c]
			1 (mgL^{-1})	20.6±4.8[b]
			10 (mgL^{-1})	90±4.8[a]
			100 (mgL^{-1})	100±4.8[a]
			1,000 (mgL^{-1})	100±4.8[a]
			10,000 (mgL^{-1})	100±4.8[a]
			100,000 (mgL^{-1})	100±4.8[a]
	Topical	Mite	0 (µg/mite)	0[d]
			0.0000117 (µg/mite)	37.5±2[c]
			0.000117 (µg/mite)	67.5±2[b]
			0.00117 (µg/mite)	100±2[a]
			0.0117 (µg/mite)	100±2[a]
			0.117 (µg/mite)	100±2[a]
			1.17 (µg/mite)	100±2[a]
	Surface contact	Bee	0 (mgL^{-1})	2.5±5.3[b]
			0.01 (mgL^{-1})	10±5.3[b]
			1 (mgL^{-1})	0[b]
			10 (mgL^{-1})	11.1±5.3[b]
			100 (mgL^{-1})	12.9±5.3[b]
			1,000 (mgL^{-1})	100±5.3[a]
			10,000 (mgL^{-1})	100±5.3[a]
	Topical	Bee	0 (µg/bee)	1±2[b]
			0.000078 (µg/bee)	1.1±2[b]
			0.00078 (µg/bee)	3.1±2[b]
			0.0078 (µg/bee)	1.9±2[b]
			0.078 (µg/bee)	2.8±2[b]
			0.78 (µg/bee)	1±2[b]
			7.8 (µg/bee)	91.5±2[a]

Classification: Public

Supplementary Table S4: Mean (±SE) mite and bee mortality (%) during 24 h exposure to different dilutions of Mitaban and control in acute surface contact (glass vial or Mason jar) or topical (micro-applicator) toxicity assays. Means with the same letter among dilations for each assay are not significantly different (*p*>0.05).

Product	Toxicity assay	Mite/bee	Dilution	Mean mortality ± SE (%)
Mitaban	Surface contact	Mite	0 (mgL^{-1})	5±5.2[c]
			1 (mgL^{-1})	7.1±5.2[c]
			10 (mgL^{-1})	16±5.2[bc]
			100 (mgL^{-1})	15.8±5.2[bc]
			1,000 (mgL^{-1})	32.8±5.2[ab]
			10,000 (mgL^{-1})	65.1±5.2[a]
	Surface contact	Bee	0 (mgL^{-1})	2.5±4.2[c]
			1 (mgL^{-1})	75.7±3.8[b]
			10 (mgL^{-1})	75.8±3.8[b]
			100 (mgL^{-1})	87.3±3.8[ab]
			1,000 (mgL^{-1})	100±3.8[a]
			10,000 (mgL^{-1})	100±3.8[a]
			100,000 (mgL^{-1})	100±3.8[a]
	Topical	Bee	0 (µg/bee)	11±4.3[a]
			0.000780 (µg/bee)	3.1±4.3[a]
			0.00780 (µg/bee)	3.9±4.3[a]
			0.0780 (µg/bee)	4.1±4.3[a]
			0.780 (µg/bee)	4.2±4.3[a]
			7.80 (µg/bee)	3.2±4.3[a]
			780 (µg/bee)	28.3±4.3[a]

Classification: Public
